# A direct method for the *N*-tetraalkylation of azamacrocycles

**DOI:** 10.3762/bjoc.12.239

**Published:** 2016-11-18

**Authors:** Andrew J Counsell, Angus T Jones, Matthew H Todd, Peter J Rutledge

**Affiliations:** 1 School of Chemistry, The University of Sydney, Sydney, New South Wales 2006, Australia

**Keywords:** azamacrocycles, biphasic system, cyclam, cyclen, *N*-alkylation

## Abstract

An efficient protocol for the direct synthesis of *N*-tetraalkylated derivatives of the azamacrocycles cyclam and cyclen has been developed, using a partially miscible aqueous–organic solvent system with propargyl bromide, benzyl bromide, and related halides. The method works most effectively when the reaction mixture is shaken, not stirred. A crystal structure of the *N*-tetrapropargyl cyclam derivative 1,4,8,11-tetra(prop-2-yn-1-yl)-1,4,8,11-tetraazacyclotetradecane diperchlorate is reported.

## Findings

The tetraazamacrocycles cyclam (**1**) and cyclen (**2**) have been the subject of considerable interest over many years [1–7]. This is due in part to the versatility of these macrocycles towards *N*-functionalisation, in part to the tendency of functionalised cyclams and cyclens to coordinate selectively a wide range of metal cations, and in part to the diverse and interesting physical and chemical behaviour displayed by the resulting complexes [2]. These systems typically exhibit high thermodynamic and kinetic stability and have found application within biomimetic systems [1,7–10], in biomedicine [3,6,11–16], as catalysts [17–22], and as fluorescent probes [5,23–28].

*N*-Tetraalkylation is a simple modification which can facilitate significant diversification of the macrocycle, often inducing marked changes to the chelation properties of the ligand [2,29]. From the first reports of *N*-tetramethylcyclam (1,4,8,11-tetramethyl-1,4,8,11-tetraazacyclotetradecane) [29–30], the simplest *N*-tetraalkyl cyclam derivative, this class of compounds has been extensively investigated [2]. While the *N*-tetramethyl derivative is readily accessed by treating cyclam with formaldehyde and formic acid [29], this transformation (the Eschweiler–Clarke methylation) [31] is not readily generalizable. Considerable effort has been invested into the development of synthetic routes to a range of *N*-tetraalkyl derivatives [2,32]. Direct synthetic approaches which use an alkyl halide and a base are highly sensitive to conditions, and tend to exhibit low yields due to the formation of quaternary amine species. A number of alternative approaches have been investigated, with differing degrees of success. For example Alcock et al. deployed a two-step route using acid chlorides to first form an intermediate amide, and then reduced this with borane in THF to the tertiary amine; yields were reasonable (71% for the tetra *n*-propyl derivative, 50% for tetra-*n*-dodecyl), but the scope was not explored beyond these two examples [33]. Fricke et al. have generated cyclen derivatives *N*-tetraalkylated with long hydrocarbon chains (unbranched C_8_, C_12_ and C_18_ alkyl chains), using *n*-BuLi to irreversibly deprotonate the macrocyclic amine NHs then introducing the appropriate alkyl bromide; however yields were low (35–40%) and the scope limited (in part by the harsh reaction conditions) [34]. Trabaud et al. have prepared a series of *N*-tetraalkyl ester and carboxylic acid derivatives using bromoester electrophiles and excess sodium bicarbonate in anhydrous DMF, but the yields were generally poor (60% in the privileged case of ethyl bromoacetate, 25–40% in all other examples) [35].

Some success has been reported using direct alkylation in biphasic solvent systems which combine an alkaline aqueous phase with an immiscible organic phase [36–38]. Tsukube used a chloroform/aqueous sodium hydroxide solvent system to generate *N*-tetrabenzylcyclam in good yield (80%), and also the *N*-benzoyl analogue [36–37]; while Alcock et al. combined dichloromethane and aqueous sodium hydroxide to make *N*-tetra-2-pyridylmethyl substituted cyclam [38]. In a related approach, Jeong and co-workers have developed an effective heterogenous system that combines a chloroform solution of cyclam and the alkyl halide with solid potassium carbonate (acknowledging the risk of competing carbene formation under these conditions), to *N*-tetraalkylate cyclam efficiently with small hydrocarbon chains (C_2_–C_4_) and also prepare the tetramethoxyethyl and -ethoxyethyl derivatives, all in excellent yields (83–90%) [39].

We sought a simple route to cyclam and cyclen derivatives *N*-tetraalkylated with propargyl (CH_2_C≡CH) and aromatic groups, as part of ongoing efforts to develop multi-functionalised cyclam derivatives for fluorescence sensing [25–28,40–41] and other biomedical applications [14–16,42–43]. However, we found the previously reported methods to be at best capricious when it comes to substitution with propargyl groups, and the other larger, hydrophobic moieties in which we are interested, leading primarily to over-alkylation and the formation of quaternary amine salts. Herein we report an efficient alternative method for the *N*-tetraalkylation of cyclam (**1**) and cyclen (**2**) in high yield, using a partially miscible aqueous-organic solvent system.

**Scheme 1:**
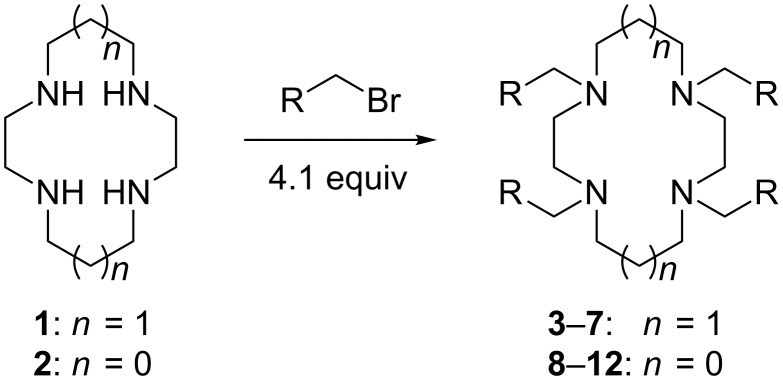
*N*-Tetraalkylation of cyclam (**1**) and cyclen (**2**) with alkyl halides in partially miscible aqueous–organic biphasic systems. Reagents and conditions: H_2_O/CH_3_CN (1:1), NaOH (1 M), RCH_2_Br (4.1 equivalents), rt, 6 h; R = C≡CH (**3** and **8**), C_6_H_5_ (**4** and **9**), *o*-bromophenyl (**5** and **10**), *p*-nitrophenyl (**6** and **11**), 2-naphthyl (**7** and **12**, see Table 1 for yields.).

The procedure combines the macrocycle and alkyl halide (4.1 equivalents), dissolved in a 1:1 mixture of aqueous sodium hydroxide solution (1 M) and CH_3_CN (Scheme 1). The mixture is then shaken, not stirred. A fine precipitate can be seen at the solvent phase barrier within five minutes, and after shaking for six hours, the precipitated product can be easily collected via filtration and washed, affording the *N*-tetraalkylated product in excellent yield and high purity.

Given our interest in triazolylcyclam derivatives, initial efforts were focused on the tetrapropargyl cyclam derivative **3**. Preparation of **3** was initially attempted via the direct alkylation of **1** (0.5 mmol) with excess propargyl bromide (6 equivalents) in acetonitrile and sodium carbonate, adapting the Trabaud protocol described above [35]. The desired compound was isolated in low yield (3%), with high levels of over-alkylation to quaternary amine salts observed. Using exactly four equivalents of the electrophile increased the yield to 30%, while adopting a modified Finkelstein procedure by adding catalytic sodium iodide gave a modest further increase in yield, to 39%. Adapting the Tsukube conditions (immiscible chloroform/sodium hydroxide solvent system [36–37]) gave a further increase in yield (to 60%), but the reaction under these conditions required long run times (up to several days) and afforded poor scalability, with the yield dropping to 42% when the reaction was carried out on a 2.5 mmol scale.

However, the synthesis of **3** was found to proceed smoothly when propargyl bromide and cyclam were combined in a mixture of aqueous sodium hydroxide and acetonitrile (Scheme 1). As noted above, a fine precipitate of **3** began to form at the phase interface within five minutes, and complete conversion had occurred after six hours. The yield and purity of **3** were found to increase significantly when the mixture was shaken and not stirred. After shaking for six hours on a rotary shaker, the crystalline precipitate was easily isolated via filtration in good yield (74%, compared with 52% when stirred conventionally with a magnetic stirrer). ^1^H NMR and elemental analysis indicated that no further purification of the isolated product was required. The percentage yield was maintained upon increasing the scale of reaction to 5 mmol of cyclam. Extending the reaction time beyond 6 hours was found to offer no further increase in yield, while performing the reaction in 100% aqueous solvent returned a lower yield (57%).

Preferential outcomes when shaking rather than stirring have been reported for other transformations, for example in building heterocycles [44] and controlling dichromate oxidation of primary alcohols to aldehydes [45], although little explanation of these observations has been put forward. Shaking reaction media is also well established as a preferred method of sample agitation in biotechnology, where it allows additional parameters (e.g., oxygen supply and hydromechanical stress) to be optimised alongside mixing performance [46], and in nanotechnology, where shaking can be used to modulate aggregation so as to favour particular nanostructures [47–48].

Otto and co-workers have investigated the influence of mechanical forces on reactivity in the context of self-replication by peptide-based disulfide macrocycles in a dynamic combinatorial library [49]. They observed different product distributions when reaction mixtures were shaken than when stirred, concluding that “mechanical forces can ... determine the outcome of a covalent synthesis” and that the ‘mechanosensitivity’ of their system is controlled by the influence of mechanical motion on nanostructure formation (the assembly of β-sheet-based fibres from the peptide building blocks in this example). Given the rapid precipitation of **3** at the phase interface observed in our *N*-tetrapropargylation reaction, it seems plausible that mechanical motion is influencing nanostructure formation and thence reaction outcomes in this context too, when shaking versus stirring the system. Alternatively the difference may be due to cavitation effects at play in the shaken system [50]. However, further work is required to determine the origins of the differential ‘shaken versus stirred’ outcomes observed in this *N*-tetraalkylation reaction.

To test the generality of the *N*-alkylation protocol, it was adapted to the preparation of a range of cyclam and cyclen derivatives, **4**–**12** (Scheme 1 and Table 1). The protocol proved effective in achieving *N*-alkylation with a range of substituted aromatic groups, affording the *N*-tetraalkyl products in generally good to excellent yields. In some instances the products were found to be partially soluble in the biphasic solvent system, which made isolation slightly less straightforward. Nonetheless, these products could be recovered from the filtrate with a simple extraction and passage through a short silica plug. Several of the products required further purification beyond the simple filtration detailed above for the *N*-tetrapropargyl cyclam derivative; this was achieved in each case either via recrystallization or by passing the product through a short silica plug (see Table 1 and Supporting Information File 1 for details).

**Table 1:** Direct synthesis of *N*-tetraalkylated macrocycles **3**–**12** from cyclam (**1**) and cyclen (**2**).

	Cyclam (**1**)		Cyclen (**2**)	
Electrophile	Product	Yield [%]	Product	Yield [%]

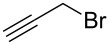	**3**	74	**8**	70^a^
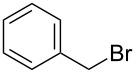	**4**	71^b^	**9**	89
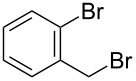	**5**	53^b^	**10**	80
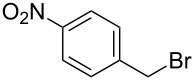	**6**	71^b^	**11**	89^c^
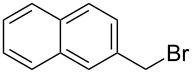	**7**	91^b^	**12**	94

^a^Purified by passage through a short silica plug; ^b^recrystallised; ^c^purified by column chromatography. See Supporting Information File 1 for details.

**Figure 1:**
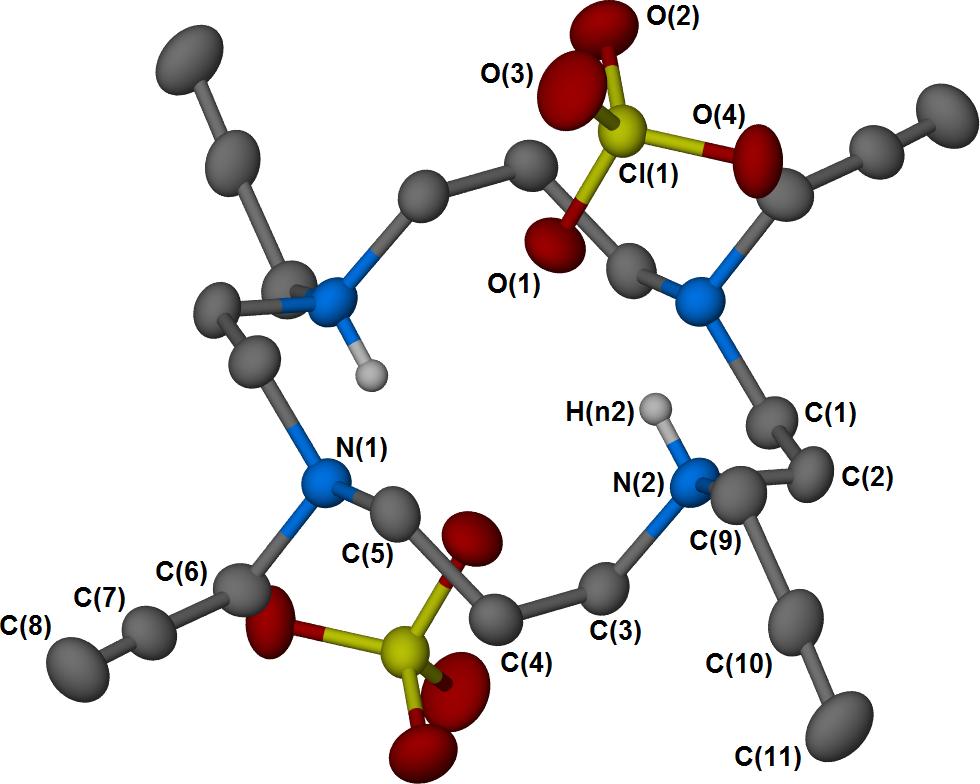
Ball-and-stick depiction of the crystal structure obtained for [(**3**)H_2_](ClO_4_)_2_, generated with X-Seed [51] and POV-Ray [52]. Ellipsoids shown at 50% probability. The asymmetric unit consists of one half of the ligand and one perchlorate anion.

Recrystallisation of **3** from a methanolic solution containing KClO_4_ yielded large, colourless, crystalline prisms. Single crystal X-ray structure determination confirmed the structure as [(**3**)H_2_](ClO_4_)_2_ (Figure 1). The asymmetric unit consists of half of the organic fragment and one perchlorate anion. Both sets of 1,4 *N*-substituents are orientated mutually *cis* to one another, with one nitrogen in the asymmetric unit protonated. Hydrogen bonding is exhibited between the oxygen of the perchlorate anion and the hydrogen of the protonated amine, with an O–H distance of 2.24(2) Å, and N–H distance of 0.87(2) Å, for a combined D–A distance of 3.014(2) Å. The protonated nitrogen atoms exhibit slightly longer N–C bond lengths (1.507(2) and 1.515(2) Å) than those of the tertiary amines (1.467(2) and 1.476(2) Å), resulting in a slight distortion of the ring and nitrogen plane.

## Conclusion

We have described an operationally simple synthetic procedure for the *N*-tetraalkylation of cyclam (**1**) and cyclen (**2**) with a range of hydrophobic functional groups. This procedure affords efficient and selective syntheses of *N*-tetraalkyl cyclams and offers several advantages over previously reported procedures. It simply requires shaking a mixed aqueous/acetonitrile solution at room temperature for six hours, i.e., no need for heating, protracted reaction times, or moisture-sensitive reagents. A further advantage of the acetonitrile/water solvent system is that for products incorporating hydrophobic side chains, the *N*-tetraalkylated species tend to precipitate almost exclusively – i.e., without contamination by mono-, di-, tri- or over-alkylated species – and in most cases little or no purification is required beyond simply collecting the precipitated product by filtration. This solvent combination facilitates complete conversion to the *N*-tetraalkyl products, and the solvent ratio can be tweaked to optimise the yield depending on the particular solubility profile of a given product. Utilisation of the *N*-tetrapropargyl products **3** and **8** to generate ‘click’-triazolylcyclam/cyclen derivatives for fluorescence sensing and other applications is underway and will be reported in due course.

## Supporting Information

File 1 Experimental procedures and characterisation data for all compounds, crystallographic information and copies of ^1^H and ^13^C NMR spectra for novel compounds.

File 2 CIF file of [**3**(H_2_)](ClO_4_)_2_, CCDC 1503283.
