# Supplementary material for: A direct method for the N-tetraalkylation of azamacrocycles
Source: Beilstein J Org Chem. 2016 Nov 18;12:2457–61. doi: 10.3762/bjoc.12.239 (PMC5238601; doi:10.3762/bjoc.12.239)
Supplement: File 1 — Experimental procedures and characterisation data for all compounds, crystallographic information and copies of 1H and 13C NMR spectra for novel compounds. [file Beilstein_J_Org_Chem-12-2457-s001.pdf]

Supporting Information

for

A direct method for the *N*-tetraalkylation of azamacrocycles

Andrew J. Counsell, Angus T. Jones, Matthew H. Todd*§ and Peter J. Rutledge*¶

Address: School of Chemistry, The University of Sydney, Sydney, New South Wales 2006, Australia

* Corresponding author

Email: Matthew H. Todd - matthew.todd@sydney.edu.au; Peter J. Rutledge - peter.rutledge@sydney.edu.au

§Tel.: +61 2 9351 2180
¶Tel.: +61 2 9351 5020

Experimental procedures and characterisation data for all compounds, crystallographic information and copies of ^{1}H and ^{13}C NMR spectra for novel compounds

Contents

1. General experimental S1

2. Synthesis and characterisation S3

3. Crystallographic data for [**3**(H_2)](ClO_4)$_2$ S8

4. References S16

5. ^{1}H and ^{13}C NMR spectra of novel compounds S17

1. General experimental

All reactions were performed in ordinary glassware. Shaking was conducted on a Vibrax® rotary shaker with medium to high revolutions per minute. All reagents and solvents were purchased from Sigma-Aldrich, Alfa Aesar, Matrix Scientific, Merck, or Ajax Finechem. Chemicals were used as received unless otherwise specified. Dichloromethane was distilled over calcium hydride prior to use. Chloroform was passed through a basic alumina column and stored over activated 4 Å molecular sieves. Acetonitrile, methanol and tetrahydrofuran were collected from a PureSolv MD 7 solvent purification system fitted with anhydrous alumina columns.

For the monitoring of reactions, analytical TLC was performed on Merck TLC Silica Gel 60 F254 (0.2 mm on aluminium). Ninhydrin stain was used to visualise amines. Flash column chromatography was performed on Merck silica gel 60 (40–63 mm), under a positive pressure of N_2 gas to optimise solvent flow.

^{1}H and ^{13}C NMR spectra were obtained on either a Bruker AVANCE DPX200 (^{1}H at 200.13 MHz, ^{13}C at 50.32 MHz), DPX300 (^{1}H at 300.13 MHz, ^{13}C at 75.47 MHz), or DRX400 (^{1}H at 400.13 MHz, ^{13}C at 100.61 MHz). Chemical shifts (δ) are reported in ppm relative to either an internal standard (0.03% v/v TMS) or the nondeuterated residual solvent peak. Coupling constants (*J*) are reported in Hertz (Hz). Signal multiplicities are reported with the following abbreviations: s - singlet, d - doublet, t - triplet, q - quartet, dd - doublet of doublets, dt - doublet of triplets, m - multiplet, br - broad. UV–vis spectra were obtained on a Varian Cary 4000 UV–vis spectrophotometer, with temperature controlled by a Varian Cary PCB water peltier system. Attenuated total reflectance (ATR) infrared spectra were recorded on a Bruker Alpha-E FT-IR spectrometer. Unless a solvent is indicated, samples were analysed as solids. Low-resolution mass spectrometry was conducted on a Finnigan LCQ Mass Spectrometer. High-resolution mass spectra were obtained on a Bruker Apex 7T Fourier Transform Ion Cyclotron Resonance (FT-ICR) Mass Spectrometer. Ionisation of samples was achieved using positive Electron Spray Ionisation (ESI). Melting points were recorded on an Optimelt Automated Melting Point System from Stanford Research Systems. Elemental analyses were performed by the Campbell Microanalytical Laboratory at the University of Otago, New Zealand.

Single crystal X-ray diffraction data was collected on an Agilent SuperNova equipped with an Atlas CCD. The crystal was harvested from amongst the diffusion supernatant, and affixed to a thin mohair fibre attached to a goniometer head with Exxon Paratone N. The crystal was quenched in a continuous stream of dry N_2 regulated by an Oxford Cryosystems Crysostream at 150(2) K. Mirror monochromated Cu-Kα radiation from a micro-source was used for data collection. Data reduction and finalisation was conducted with CrysAlisPro [1]. Further computations were undertaken within the WinGX [2] graphical user interface. Structures were solved by direct methods with either SIR97 [3] or SHELXS-2013 [4]. Structures were refined with SHELXL-2016 [4] using the full-matrix least-squares on F^2 method. All non-hydrogen atoms in main residues were modelled with anisotropic displacement parameters, and a riding atom model applied for hydrogen atoms. Hydrogen atoms taking part in a hydrogen bonding network were located in final difference maps and modelled with an isotropic displacement parameter. Hydrogen atoms from solvent residues not partaking in a hydrogen bonding network were not modelled. Structure analysis and visualisation were carried out in POV-Ray [5], Mercury 3.3 [6], X-Seed [7], and PLATON [8].

2. Synthesis and characterisation

General synthetic procedure A: *N-tetraalkylation of macrocycles*

Macrocycle (1.0 equiv) and alkyl halide (4.1 equiv) were dissolved in a 1:1 mixture of aqueous 1 M NaOH and CH_3CN (5–20 mL). The reaction mixture was shaken for 6 h. The resulting precipitate was collected by filtration, washed with hexane, and dried in vacuo to give the desired *N*-tetraalkyl derivative.

1,4,8,11-Tetra(prop-2-yn-1-yl)-1,4,8,11-tetraazacyclotetradecane (3)

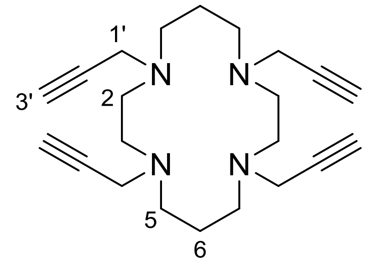

Cyclam (1.00 g, 4.99 mmol) and propargyl bromide (80% in toluene, 2.20 mL, 20.4 mmol) were reacted according to general synthetic procedure A to yield **3** as white prismodic crystals (1.31 g, 3.69 mmol, 74%). **m.p.** 135–137°C (no lit. m.p.). **IR** ν_{max}/cm^{-1} 3271, 3170, 2815, 2091, 1453, 1433, 1369, 1126, 1078, 990, 795, 748, 689, 649, 621, 554. **^{1}H NMR** ($CDCl_3$, 300 MHz): δ 1.59 (4H, qn, *J* 6.6, H^6), 2.16 (4H, t, *J* 2.2, $H^{3'}$), 2.55–2.61 (16H, m, H^2 and H^5), 3.43 (8H, d, *J* 2.4, $H^{1'}$). **^{13}C NMR** (75 MHz): δ 24.8, 42.6, 49.8, 50.0, 72.9, 78.6. **LRMS** (ESI+) *m/z* 353.2 ([M+H]$^+$, 100%). **HRMS** (ESI+) 353.26997 [M+H]$^+$; calculated for $C_{22}H_{33}N_4$ [M+H]$^+$ 353.26997. **Anal. Calcd.** for $C_{22}H_{32}N_4$: C 74.96, H 9.15, N 15.89. Found C 74.99, H 9.36, N 16.02.

1,4,8,11-Tetrabenzyl-1,4,8,11-tetraazacyclotetradecane (4)

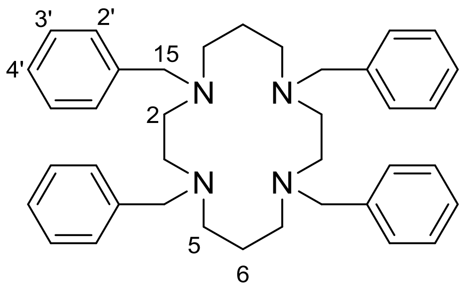

Cyclam (200 mg, 1.00 mmol) and benzyl bromide (719 mg, 4.20 mmol) were reacted according to general synthetic procedure A. The resulting precipitate was recrystallised from CH_2Cl_2:MeOH (1:1), to give **4** as clear colourless prisms (340 mg, 71%). **m.p.** 151–153°C (lit. [9] 151–153°C). **^{1}H NMR** ($CDCl_3$, 300 MHz): δ 1.79 (8H, m, H^6), 2.55 (8H, t, *J* 7.5, H^5), 2.63 (8H, s, H^2), 3.47 (8H, s, H^{15}),

7.10–7.40 (20H, m, $H^{2'}$–$H^{4'}$). **LRMS** (ESI+) *m/z* 561.76 ($[M+H]^+$, 100%), 471.75 ($[M+H]^+$, 15%). Spectroscopic data match those reported in the literature [9].

1,4,8,11-Tetrakis(2-bromobenzyl)-1,4,8,11-tetraazacyclotetradecane (5)

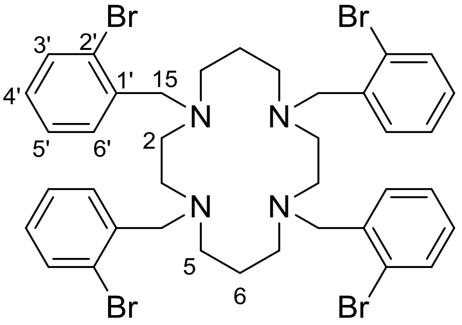

Cyclam **1** (200 mg, 1.00 mmol) and 2-bromobenzyl bromide (1.05 g, 4.20 mmol) were reacted according to general synthetic procedure A. The resulting precipitate was recrystallised from CH_2Cl_2:MeOH (1:1) to give **5** as off-white prisms (460 mg, 53%). **m.p.** 172–174 °C (no lit. m.p.). **IR** ν_{max}/cm^{-1} 3233 (br), 1632. **^{1}H NMR** ($CDCl_3$, 200 MHz): δ 1.79 (4H, qn, *J* 6.6, H^6), 2.65 (8H, t, *J* 6.6, H^5), 2.76 (8H, s, H^3), 3.62 (8H, s, H^{15}), 7.05 (8H, t, *J* 7.5, $H^{5'}$), 7.18 (4H, t, *J* 7.5, $H^{4'}$), 7.40–7.60 (8H, m, $H^{3'}$ and $H^{6'}$). **^{13}C NMR** ($CDCl_3$, 75 MHz): 23.9, 50.9, 51.6, 58.8, 124.3, 127.1, 128.1, 130.8, 132.5, 139.0. **LRMS** (ESI+) *m/z* 877.5 ($[M(^{81}Br_2{}^{79}Br_2)+H]^+$, 100%), 875.5 (85%, $[M(^{81}Br^{79}Br_3)+H]^+$, 85%), 879.4 ($[M(^{81}Br_3{}^{79}Br)+H]^+$, 78%). **HRMS** (ESI+) calcd. for $C_{38}H_{45}Br_4N_4$ ($[M(^{81}Br_2{}^{79}Br_2)+H]^+$) 873.03722, found 877.03267 ($[M(^{81}Br_2{}^{79}Br_2)+H]^+$, 100%), 875.03569 ($[M(^{81}Br^{79}Br_3)+H]^+$, 60%), 879.03030 ($[M(^{81}Br_3{}^{79}Br_1)+H]^+$, 50%); **Anal. Calcd.** for $C_{38}H_{44}Br_4N_4$: C 52.08, H 5.06, N 6.39. Found: C, 52.08; H, 5.13; N, 6.40.

1,4,8,11-Tetrakis(4-nitrobenzyl)-1,4,8,11-tetraazacyclotetradecane (6)

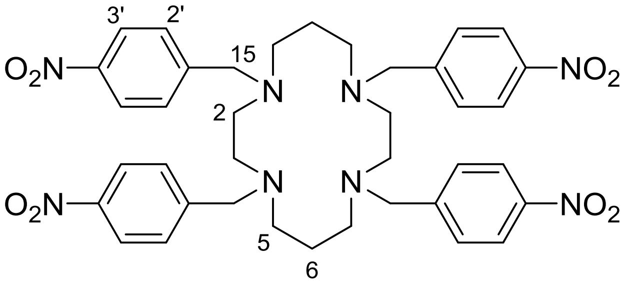

Cyclam (100 mg, 0.50 mmol) and 4-nitrobenzyl bromide (450 mg, 2.08 mmol) were reacted according to general synthetic procedure A. The resultant precipitate was recrystallised from CH_2Cl_2:hexane (9:1) to give **6** as yellow irregular fused prisms (264 mg, 71%). **m.p.** 161–163 °C (no lit. m.p.). **^{1}H NMR** ($CDCl_3$, 200 MHz): δ 1.70–1.90 (8H, m, H^6), 2.55–2.70 (16H, m, H^2 and H^5), 3.53 (8H, s, H^{15}), 7.51 (8H, d, *J* 8, $H^{2'}$), 8.15 (8H, d, *J* 8.2, $H^{3'}$). **LRMS** (ESI+) *m/z* 741.79 ($[M+H]^+$, 100%). Spectroscopic data match those reported in the literature [10].

1,4,8,11-Tetrakis(naphthalen-2-ylmethyl)-1,4,8,11-tetraazacyclotetradecane (7)

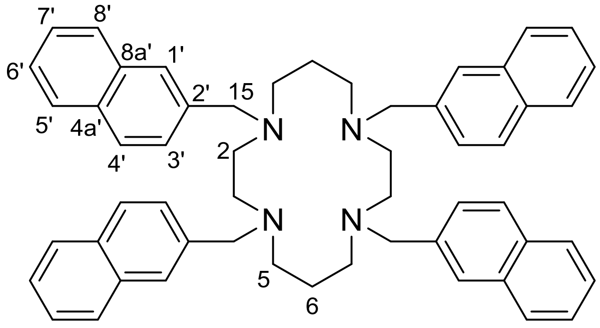

Cyclam (203 mg, 1.01 mmol) and 2-bromomethylnaphthalene (912 mg, 4.12 mmol) were reacted according to general synthetic procedure A. The resultant precipitate was recrystallised from THF to give **7** as off-white needles (703 mg, 91%). **m.p.** 202–204°C (lit. [11] m.p. 204 °C). **^{1}H NMR** ($CDCl_3$, 300 MHz): δ 1.85 (4H, m, H^6), 2.60 (8H, t, *J* 6.3, H^5), 2.70 (8H, s, H^2), 3.57 (8H, s, H^{15}), 7.20–7.90 (28H, m, $H^{1'}$ and $H^{3'}$–$H^{8'}$). **LRMS** (ESI+) *m/z* 761.87 ($[M+H]^+$, 100%). Spectroscopic data match those reported in the literature [11].

1,4,7,10-Tetra(prop-2-yn-1-yl)-1,4,7,10-tetraazacyclododecane (8)

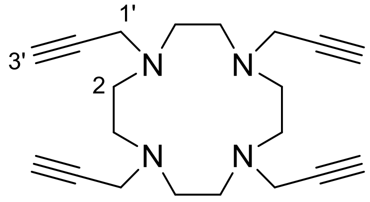

Cyclen (4.06 g, 23.6 mmol) and propargyl bromide (80% in toluene, 4.1 mL, 36 mmol) were reacted according to general synthetic procedure A. The filtrate was collected and the CH_3CN removed under reduced pressure, and the remaining product extracted with EtOAc (3 × 40 mL). The combined organic phases were washed with brine (60 mL), dried over $MgSO_4$, and concentrated under reduced pressure. The residue was taken up in EtOAc and passed through a short silica column to give **8** as a light brown crystalline solid (5.36 g, 16.5 mmol, 70% total). **m.p.** 93–95°C (lit. [12] m.p. 92°C). **^{1}H NMR** ($CDCl_3$, 300 MHz): *δ* 2.16 (4H, t, *J* 2.2, $H^{3'}$), 2.70 (16H, s, H^2), 3.44 (8H, d, *J* 2.2, $H^{1'}$). **LRMS (ESI+)** *m/z* 325.00 $[M+H]^+$. Spectroscopic data match those reported in the literature [12].

1,4,7,10-Tetrabenzyl-1,4,7,10-tetraazacyclododecane (9)

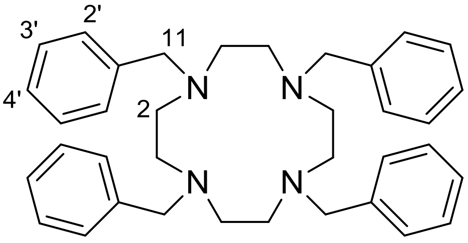

Cyclen (171 mg, 1.00 mmol) and benzyl bromide (700 mg, 4.1 mmol) were reacted according to general synthetic procedure A to yield **9** as a fine white powder (470 mg, 89%). **m.p.** 145–148°C (lit. [13] m.p. 145-147°C). **^{1}H NMR** ($CDCl_3$, 300 MHz): δ 2.68 (16H, s, H^2), 3.43 (8H, s, H^{11}), 7.15–7.40 (20H, m, $H^{2'}$-$H^{4'}$). **LRMS** (ESI+) *m/z* 533.31 ($[M+H]^+$, 100%). Spectroscopic data match those reported in the literature [13].

1,4,7,10-Tetrakis(2-bromobenzyl)-1,4,7,10-tetraazacyclododecane (10)

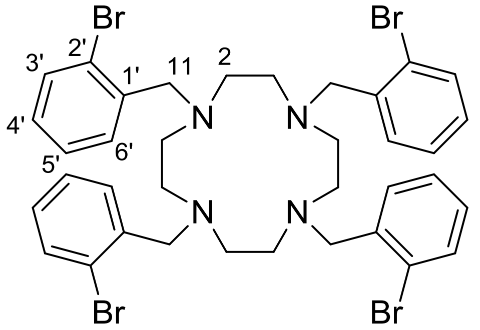

Cyclen (170.6 mg, 0.9902 mmol) and 2-bromobenzyl bromide (1.033 mg, 4.131 mmol) were reacted according to general synthetic procedure A to yield **10** as a white powder (848.36 mg, 80%). **m.p.** 116–120°C (no lit. m.p.). **IR** ν_{max}/cm^{-1} 437, 697, 731, 749, 906, 1024, 1437, 1565, 2798. **^{1}H NMR** ($CDCl_3$, 300 MHz): δ 2.78 (16H, s, H^2), 3.53 (8H, s, H^{11}), 6.85–7.92 (16H, m, $H^{3'}$–$H^{6'}$). **^{13}C NMR** (75 MHz): δ 53.9, 59.6, 123.9, 127.3 127.9, 130.6, 132.4, 139.0. **LRMS** (ESI+) *m/z* 848.94 ($[M+H]^+$, 100%). **HRMS** (ESI+) 849.00178 $[M+H]^+$; calculated for $C_{36}H_{41}Br_4N_4$ $[M+H]^+$ 849.00183.

1,4,7,10-Tetrakis(4-nitrobenzyl)-1,4,7,10-tetraazacyclododecane (11)

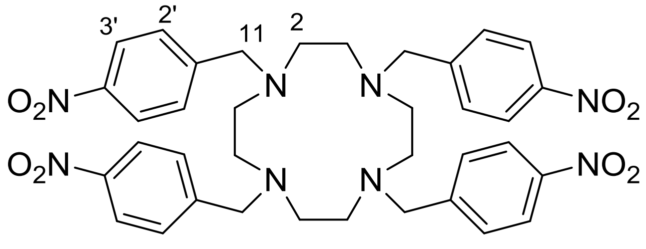

Cyclen (171.3 mg, 0.994 mmol) and p-nitrobenzyl bromide (0.886 mg, 4.10 mmol) were reacted according to general synthetic procedure A. The resultant precipitate was purified by column chromatography (CH_2Cl_2:MeOH, 1:99 ramping to 1:9, silica gel) to yield **11** as yellow powder (635 mg, 89%). **m.p.** 196–198°C (lit. [14] m.p. 193-194°C). **^{1}H NMR** ($CDCl_3$, 300 MHz): δ 2.71 (16H, s, H^2), 3.53 (8H, s, H^{11}), 7.45–7.55 (8H, m, $H^{2'}$) 8.05–8.15 (8H, m, $H^{3'}$). **LRMS** (ESI+) *m/z* 713.29 ($[M+H]^+$, 100%). Spectroscopic data match those reported in the literature [15].

1,4,7,10-Tetrakis(naphthalen-2-ylmethyl)-1,4,7,10-tetraazacyclododecane (12)

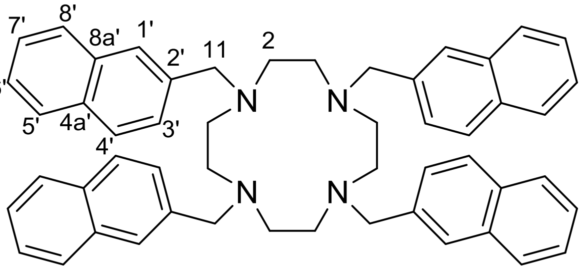

Cyclen (171.6 mg, 0.996 mmol) and 2-bromomethylnaphthalene (890 mg, 4.02 mmol) were reacted according to general synthetic procedure A to yield **12** as a fine white powder (684 mg, 94%). **m.p.** 116–120°C (no lit. m.p.)**. IR** ν_{max}/cm^{-1} 471, 731, 747, 799, 821, 853, 1078, 1286, 1351, 1446, 1505, 2802. **^{1}H NMR** ($CDCl_3$, 300 MHz): δ 2.79 (16H, s, H^2), 3.57 (8H, s, H^{11}), 7.20–7.90 (28H, m, $H^{1'}$ and $H^{3'}$–$H^{8'}$). **^{13}C NMR** (75 MHz): δ 53.1, 60.6, 125.4, 125.8 127.4, 127.8, 132.8, 133.4, 137.9. **LRMS** (ESI+) *m/z* 733.39 ($[M+H]^+$, 100%). **HRMS** (ESI+) 733.42562 $[M+H]^+$; calculated for $C_{52}H_{52}N_4$ $[M+H]^+$ 733.42647.

3. Crystallographic data for [3(H_2)](ClO_4)$_2$

Table S1 - Crystal data and structure refinement for salt [**3**(H_2)](ClO_4)$_2$

Empirical formula	C22 H34 Cl2 N4 O8	
Formula weight	553.43	
Temperature	150(2) K	
Wavelength	1.54178 Å	
Crystal system	Monoclinic	
Space group	P 21/n	
Unit cell dimensions	a = 9.7218(3) Å	α = 90°.
	b = 14.1376(2) Å	β = 115.650(3)°.
	c = 10.2523(2) Å	γ = 90°.
Volume	1270.25(6) Å^3	
Z	2	
Density (calculated)	1.447 Mg/m^3	
Absorption coefficient	2.772 mm^{-1}	
F(000)	584	
Crystal size	0.05 x 0.04 x 0.01 mm^3	
Theta range for data collection	3.1259 to 76.1941°.	
Index ranges	-12<=h<=12, -17<=k<=17, -12<=l<=12	
Reflections collected	37188	
Independent reflections	2646 [R(int) = 0.0598]	
Completeness to theta = 74.3338°	99.89 %	
Absorption correction	Semi-empirical from equivalents	
Max. and min. transmission	1.00000 and 0.82343	
Refinement method	Full-matrix least-squares on F^2	
Data / restraints / parameters	2646 / 0 / 168	
Goodness-of-fit on F^2	1.058	
Final R indices [I>2sigma(I)]	R1 = 0.0343, wR2 = 0.0869	
R indices (all data)	R1 = 0.0410, wR2 = 0.0909	
Largest diff. peak and hole	0.260 and -0.483 e.Å^{-3}	

Table S2 - Atomic coordinates (x 10^4) and equivalent isotropic displacement parameters ($Å^2 \times 10^3$) for [**3**(H_2)](ClO_4)$_2$. U(eq) is defined as one third of the trace of the orthogonalized U^{ij} tensor.

	x	y	z	U(eq)
C(1)	1731(2)	18(1)	-1464(2)	24(1)
C(2)	1836(2)	-68(1)	-2894(2)	24(1)
C(3)	4190(2)	905(1)	-2202(2)	24(1)
C(4)	5751(2)	1031(1)	-2183(2)	26(1)
C(5)	7042(2)	453(1)	-1054(2)	23(1)
C(6)	7950(2)	1655(1)	831(2)	25(1)
C(7)	9350(2)	1822(1)	652(2)	27(1)
C(8)	10453(2)	1955(1)	449(2)	35(1)
C(9)	3590(2)	-434(1)	-4001(2)	26(1)
C(10)	2940(2)	243(1)	-5193(2)	29(1)
C(11)	2434(2)	807(1)	-6129(2)	38(1)
N(1)	7307(1)	695(1)	432(1)	21(1)
N(2)	3480(2)	-64(1)	-2671(1)	22(1)
O(1)	4349(2)	1646(1)	1119(1)	34(1)
O(2)	4888(2)	3011(1)	122(1)	40(1)
O(3)	3662(2)	3145(1)	1640(2)	43(1)
O(4)	6246(1)	2690(1)	2614(1)	34(1)
CL1	4784(1)	2631(1)	1381(1)	24(1)

Table S3 - Bond lengths [Å] and angles [°] for [**3**(H_2)](ClO_4)$_2$.

C(1)-N(1)#1	1.4674(18)
C(1)-C(2)	1.518(2)
C(1)-H(1A)	0.9700
C(1)-H(1B)	0.9700
C(2)-N(2)	1.5133(19)
C(2)-H(2B)	0.9700
C(2)-H(2A)	0.9700
C(3)-N(2)	1.5157(18)
C(3)-C(4)	1.520(2)
C(3)-H(3A)	0.9700
C(3)-H(3B)	0.9700
C(4)-C(5)	1.525(2)
C(4)-H(4A)	0.9700
C(4)-H(4B)	0.9700
C(5)-N(1)	1.4707(18)
C(5)-H(5B)	0.9700
C(5)-H(5A)	0.9700
C(6)-C(7)	1.470(2)
C(6)-N(1)	1.4760(18)
C(6)-H(6A)	0.9700
C(6)-H(6B)	0.9700
C(7)-C(8)	1.192(3)
C(8)-H(8)	0.9300
C(9)-C(10)	1.463(2)
C(9)-N(2)	1.5069(18)
C(9)-H(9A)	0.9700
C(9)-H(9B)	0.9700
C(10)-C(11)	1.179(3)
C(11)-H(11)	0.9300
N(1)-C(1)#1	1.4675(18)
N(2)-HN2	0.87(2)
O(1)-CL1	1.4462(11)
O(2)-CL1	1.4418(13)
O(3)-CL1	1.4276(13)
O(4)-CL1	1.4388(13)
N(1)#1-C(1)-C(2)	110.71(12)

N(1)#1-C(1)-H(1A)	109.5
C(2)-C(1)-H(1A)	109.5
N(1)#1-C(1)-H(1B)	109.5
C(2)-C(1)-H(1B)	109.5
H(1A)-C(1)-H(1B)	108.1
N(2)-C(2)-C(1)	111.23(12)
N(2)-C(2)-H(2B)	109.4
C(1)-C(2)-H(2B)	109.4
N(2)-C(2)-H(2A)	109.4
C(1)-C(2)-H(2A)	109.4
H(2B)-C(2)-H(2A)	108.0
N(2)-C(3)-C(4)	115.11(12)
N(2)-C(3)-H(3A)	108.5
C(4)-C(3)-H(3A)	108.5
N(2)-C(3)-H(3B)	108.5
C(4)-C(3)-H(3B)	108.5
H(3A)-C(3)-H(3B)	107.5
C(3)-C(4)-C(5)	115.88(12)
C(3)-C(4)-H(4A)	108.3
C(5)-C(4)-H(4A)	108.3
C(3)-C(4)-H(4B)	108.3
C(5)-C(4)-H(4B)	108.3
H(4A)-C(4)-H(4B)	107.4
N(1)-C(5)-C(4)	112.30(12)
N(1)-C(5)-H(5B)	109.1
C(4)-C(5)-H(5B)	109.1
N(1)-C(5)-H(5A)	109.1
C(4)-C(5)-H(5A)	109.1
H(5B)-C(5)-H(5A)	107.9
C(7)-C(6)-N(1)	114.57(12)
C(7)-C(6)-H(6A)	108.6
N(1)-C(6)-H(6A)	108.6
C(7)-C(6)-H(6B)	108.6
N(1)-C(6)-H(6B)	108.6
H(6A)-C(6)-H(6B)	107.6
C(8)-C(7)-C(6)	177.45(17)
C(7)-C(8)-H(8)	180.0
C(10)-C(9)-N(2)	110.77(12)
C(10)-C(9)-H(9A)	109.5

N(2)-C(9)-H(9A)	109.5
C(10)-C(9)-H(9B)	109.5
N(2)-C(9)-H(9B)	109.5
H(9A)-C(9)-H(9B)	108.1
C(11)-C(10)-C(9)	178.27(19)
C(10)-C(11)-H(11)	180.0
C(1)#1-N(1)-C(5)	111.00(11)
C(1)#1-N(1)-C(6)	111.74(12)
C(5)-N(1)-C(6)	111.42(11)
C(9)-N(2)-C(2)	110.23(12)
C(9)-N(2)-C(3)	113.60(11)
C(2)-N(2)-C(3)	110.93(11)
C(9)-N(2)-HN2	106.1(13)
C(2)-N(2)-HN2	106.5(13)
C(3)-N(2)-HN2	109.1(13)
O(3)-CL1-O(4)	110.56(8)
O(3)-CL1-O(2)	109.40(9)
O(4)-CL1-O(2)	109.87(8)
O(3)-CL1-O(1)	109.79(8)
O(4)-CL1-O(1)	108.48(8)
O(2)-CL1-O(1)	108.71(8)

Symmetry transformations used to generate equivalent atoms:

#1 -x+1,-y,-z

Table S4 - Anisotropic displacement parameters ($Å^2 \times 10^3$) for [**3**(H_2)](ClO_4)$_2$. The anisotropic displacement factor exponent takes the form: $-2\pi^2[h^2 a^{*2}U^{11} + ... + 2 h k a^* b^* U^{12}]$

	U^{11}	U^{22}	U^{33}	U^{23}	U^{13}	U^{12}
C(1)	22(1)	24(1)	24(1)	2(1)	9(1)	2(1)
C(2)	20(1)	28(1)	21(1)	1(1)	5(1)	0(1)
C(3)	25(1)	22(1)	21(1)	-1(1)	6(1)	1(1)
C(4)	29(1)	26(1)	22(1)	2(1)	8(1)	-5(1)
C(5)	23(1)	25(1)	21(1)	-3(1)	9(1)	-4(1)
C(6)	30(1)	21(1)	25(1)	-3(1)	13(1)	0(1)
C(7)	32(1)	23(1)	22(1)	-3(1)	9(1)	-6(1)
C(8)	36(1)	37(1)	34(1)	-1(1)	17(1)	-10(1)
C(9)	29(1)	27(1)	21(1)	-3(1)	10(1)	1(1)
C(10)	29(1)	37(1)	18(1)	-4(1)	8(1)	0(1)
C(11)	41(1)	47(1)	22(1)	4(1)	9(1)	8(1)
N(1)	21(1)	21(1)	20(1)	1(1)	8(1)	0(1)
N(2)	23(1)	23(1)	16(1)	2(1)	6(1)	2(1)
O(1)	39(1)	21(1)	35(1)	-2(1)	8(1)	-5(1)
O(2)	40(1)	42(1)	34(1)	13(1)	13(1)	0(1)
O(3)	34(1)	44(1)	49(1)	-6(1)	16(1)	13(1)
O(4)	24(1)	42(1)	30(1)	-6(1)	5(1)	-4(1)
CL1	21(1)	21(1)	25(1)	-1(1)	6(1)	0(1)

Table S5 - Hydrogen coordinates (× 10^4) and isotropic displacement parameters ($Å^2$ × 10^{3}) for [**3**(H_2)](ClO_4)$_2$.

	x	y	z	U(eq)
H(1A)	2056	645	-1065	28
H(1B)	679	-66	-1624	28
H(2B)	1350	-650	-3370	29
H(2A)	1296	456	-3517	29
H(3A)	4279	1032	-1239	32(5)
H(3B)	3504	1374	-2848	39
H(4A)	6025	1695	-2023	32
H(4B)	5672	866	-3131	32
H(5B)	6796	-214	-1220	28
H(5A)	7971	563	-1164	28
H(6A)	7185	2110	245	30
H(6B)	8165	1770	1835	30
H(8)	11313	2059	291	42
H(9A)	4652	-549	-3782	31
H(9B)	3047	-1030	-4287	31
H(11)	2035	1252	-6867	46
HN2	3960(20)	-468(14)	-1990(20)	26(5)

Table S6 - Torsion angles [°] for [**3**(H_2)](ClO_4)$_2$.

N(1)#1-C(1)-C(2)-N(2)	-52.07(16)
N(2)-C(3)-C(4)-C(5)	-67.14(17)
C(3)-C(4)-C(5)-N(1)	-61.39(17)
C(4)-C(5)-N(1)-C(1)#1	165.87(12)
C(4)-C(5)-N(1)-C(6)	-68.90(15)
C(7)-C(6)-N(1)-C(1)#1	69.99(16)
C(7)-C(6)-N(1)-C(5)	-54.83(17)
C(10)-C(9)-N(2)-C(2)	70.54(16)
C(10)-C(9)-N(2)-C(3)	-54.66(17)
C(1)-C(2)-N(2)-C(9)	163.15(12)
C(1)-C(2)-N(2)-C(3)	-70.13(15)
C(4)-C(3)-N(2)-C(9)	-45.71(17)
C(4)-C(3)-N(2)-C(2)	-170.54(12)

Symmetry transformations used to generate equivalent atoms:

#1 -x+1,-y,-z

Table S7 - Hydrogen bonds for [**3**(H_2)](ClO_4)$_2$ [Å and °].

D-H...A	d(D-H)	d(H...A)	d(D...A)	<(DHA)
C(3)-H(3A)...O(1)	0.97	2.54	3.5014(19)	170.1
C(6)-H(6A)...O(2)	0.97	2.53	3.345(2)	142.1
N(2)-HN2...O1$1#1	0.87(2)	2.24(2)	3.0139(18)	149.2(17)

Symmetry transformations used to generate equivalent atoms:

#1 -x+1,-y,-z

4. References

1. *CrysalisPRO*, Oxford Diffraction, Agilent Technologies UK Ltd: Yarnton, England.
2. Farrugia, L. *J. Appl. Crystallogr.* **2012**, *45*, 849-854.
3. Altomare, A.; Burla, M. C.; Camalli, M.; Cascarano, G. L.; Giacovazzo, C.; Guagliardi, A.; Moliterni, A. G. G.; Polidori, G.; Spagna, R. *J. Appl. Crystallogr.* **1999**, *32*, 115-119.
4. Sheldrick, G. *Acta Crystallogr. Sect. A* **2008**, *64*, 112-122.
5. *Persistence of Vision (TM) Raytracer (Version 3.6)*, Persistence of Vision Pty. Ltd.: 2004.
6. Edgington, P. R.; McCabe, P.; Macrae, C. F.; Pidcock, E.; Shields, G. P.; Taylor, R.; Towler, M.; Van De Streek, J. *J. Appl. Crystallogr.* **2006**, *39*, 453-457.
7. Barbour, L. J. *J. Supramol. Chem.* **2001**, *1*, 189-191.
8. Spek, A. *Acta Crystallogr. Sect. D* **2009**, *65*, 148-155.
9. Saudan, C.; Ceroni, P.; Vicinelli, V.; Balzani, V.; Gorka, M.; Lee, S.-K.; Vögtle, F.; Orlandi, M.; Bartolini, G.; Tavolari, S.; Rocchi, P.; Ferreri, A. M. *Supramol. Chem.* **2004**, *16*, 541-548.
10. Enoki, O.; Imaoka, T.; Yamamoto, K. *Org. Lett.* **2003**, *5*, 2547-2549.
11. Saudan, C.; Balzani, V.; Ceroni, P.; Gorka, M.; Maestri, M.; Vicinelli, V.; Vögtle, F. *Tetrahedron* **2003**, *59*, 3845-3852.
12. Xu, H.-B.; Chao, H.-Y. *Inorg. Chem. Commun.* **2007**, *10*, 1129-1131.
13. Gelmboldt, V. O.; Ganin, E. V.; Basok, S. S.; Kulygina, E. Y.; Botoshansky, M. M.; Kravtsov, V. C.; Fonari, M. S. *CrystEngComm* **2011**, *13*, 3682-3685.
14. Habata, Y.; Ikeda, M.; Yamada, S.; Takahashi, H.; Ueno, S.; Suzuki, T.; Kuwahara, S. *Org. Lett.* **2012**, *14*, 4576-4579.
15. Deng, Q.; Jin, Y.; Wang, Q.; Zhao, R.; Pan, N.; Zhai, F.; Luo, M.; Xia, C. *J. Radioanal. Nucl. Chem.* **2013**, *295*, 125-133.

5. ^{1}H and ^{13}C NMR spectra of novel compounds

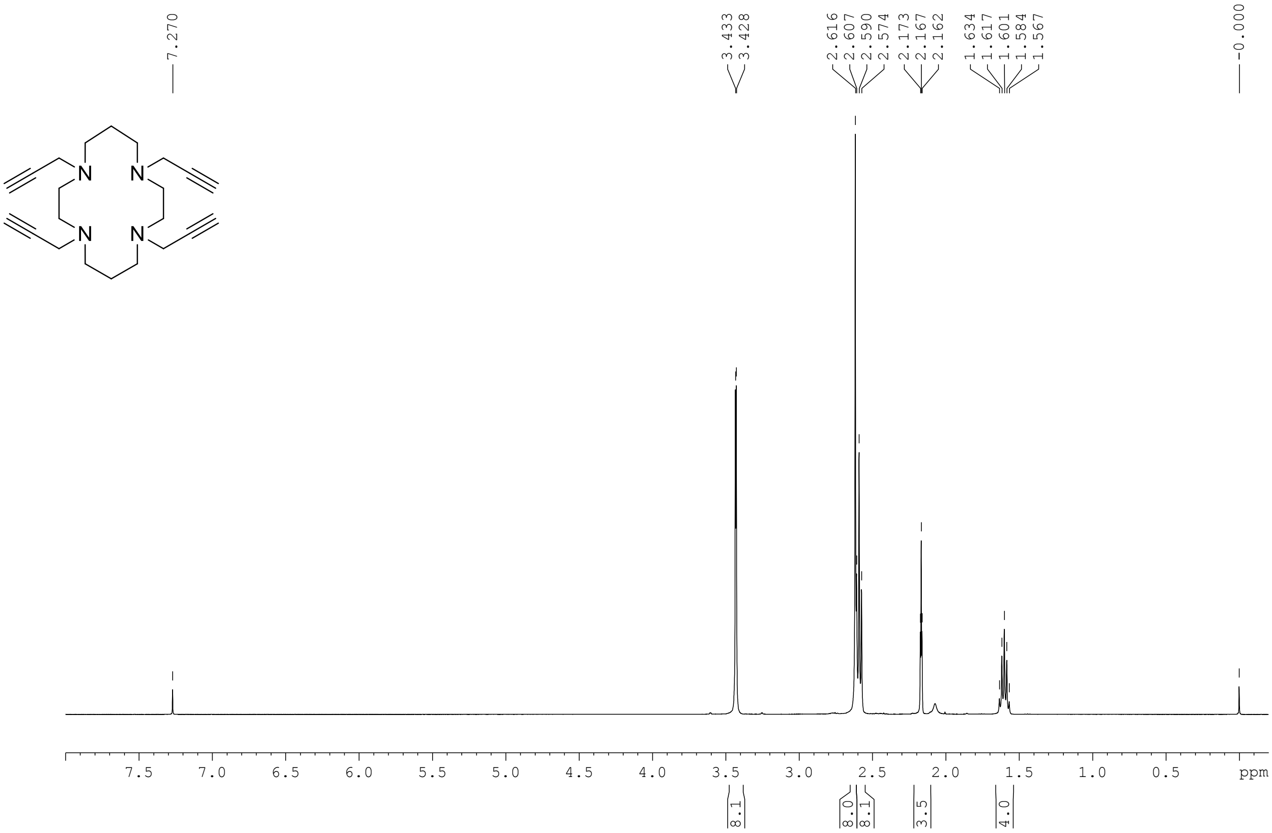

Figure S1 - ^{1}H NMR spectrum (300 MHz) of **3** in $CDCl_3$.

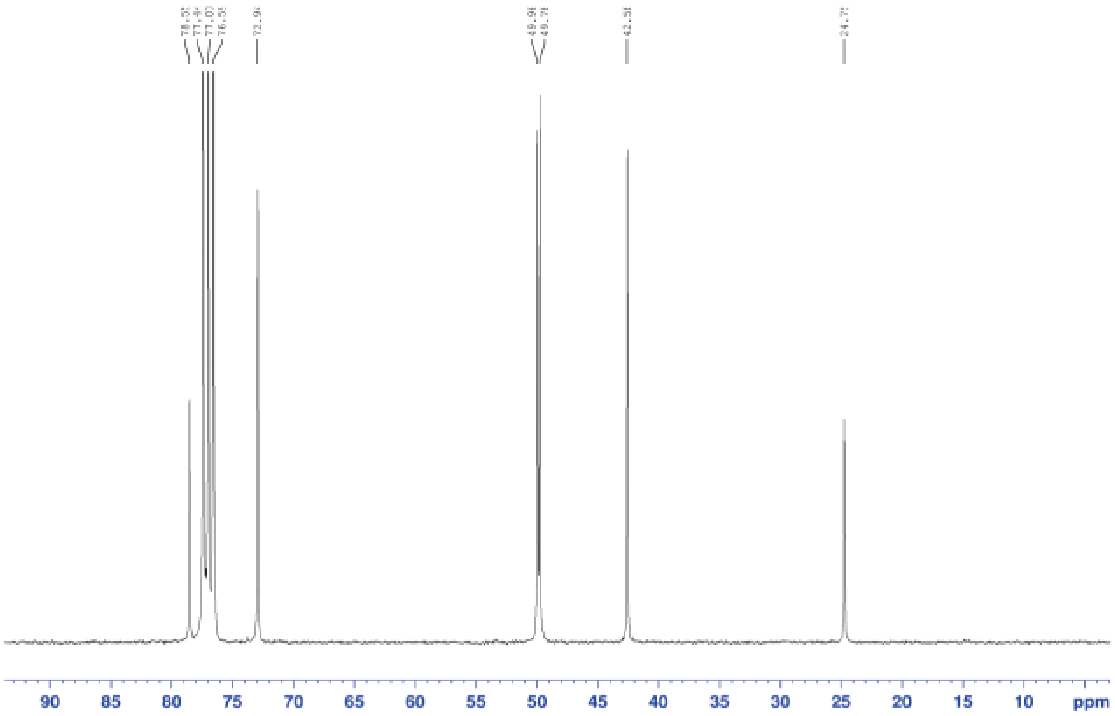

Figure S2 – ^{13}C NMR spectrum (75 MHz) of **3** in $CDCl_3$.

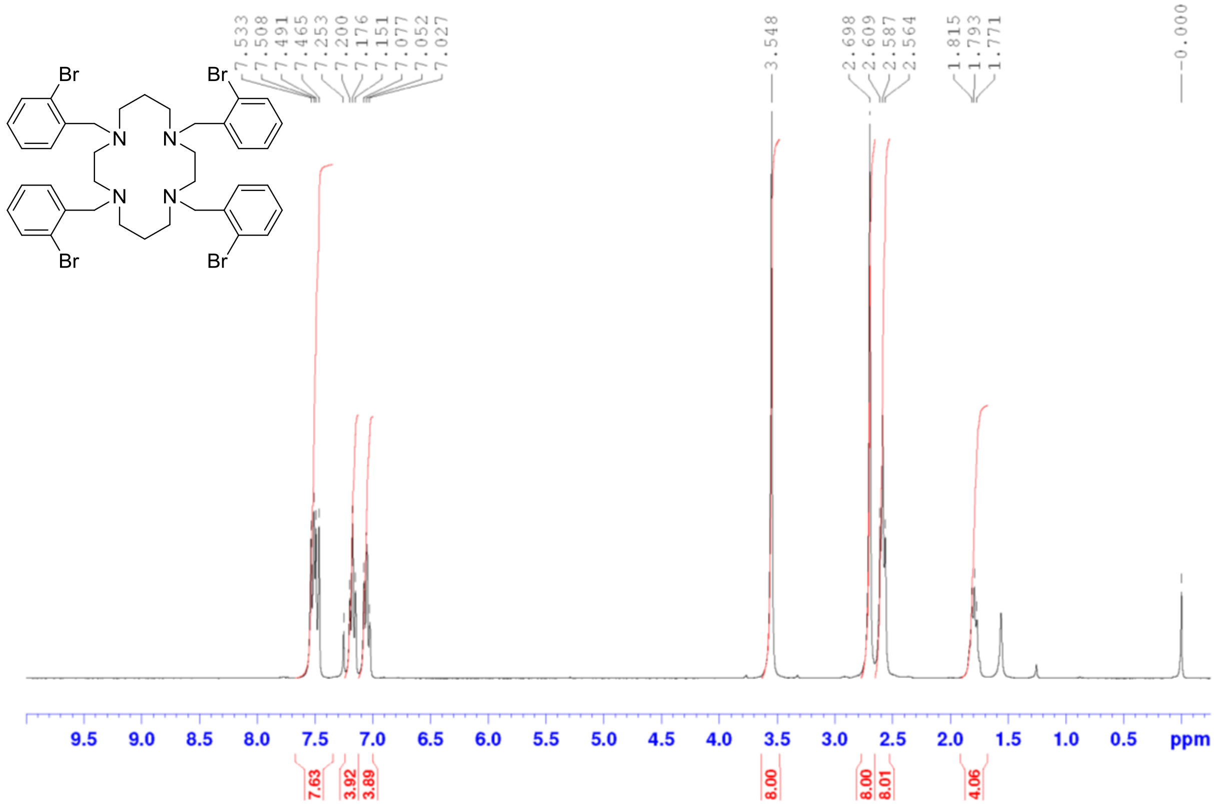

Figure S3 - ^{1}H NMR spectrum (300 MHz) of **5** in $CDCl_3$.

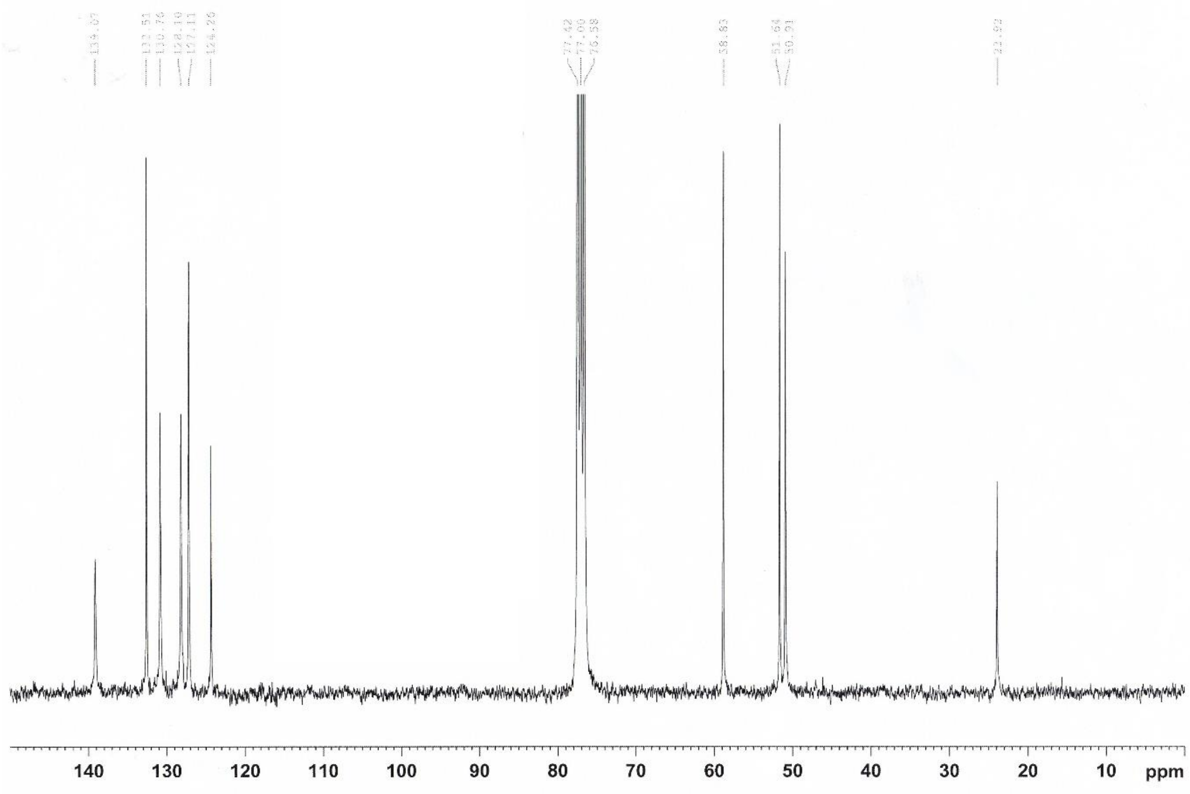

Figure S4 - ^{13}C NMR spectrum (75 MHz) of **5** in $CDCl_3$.

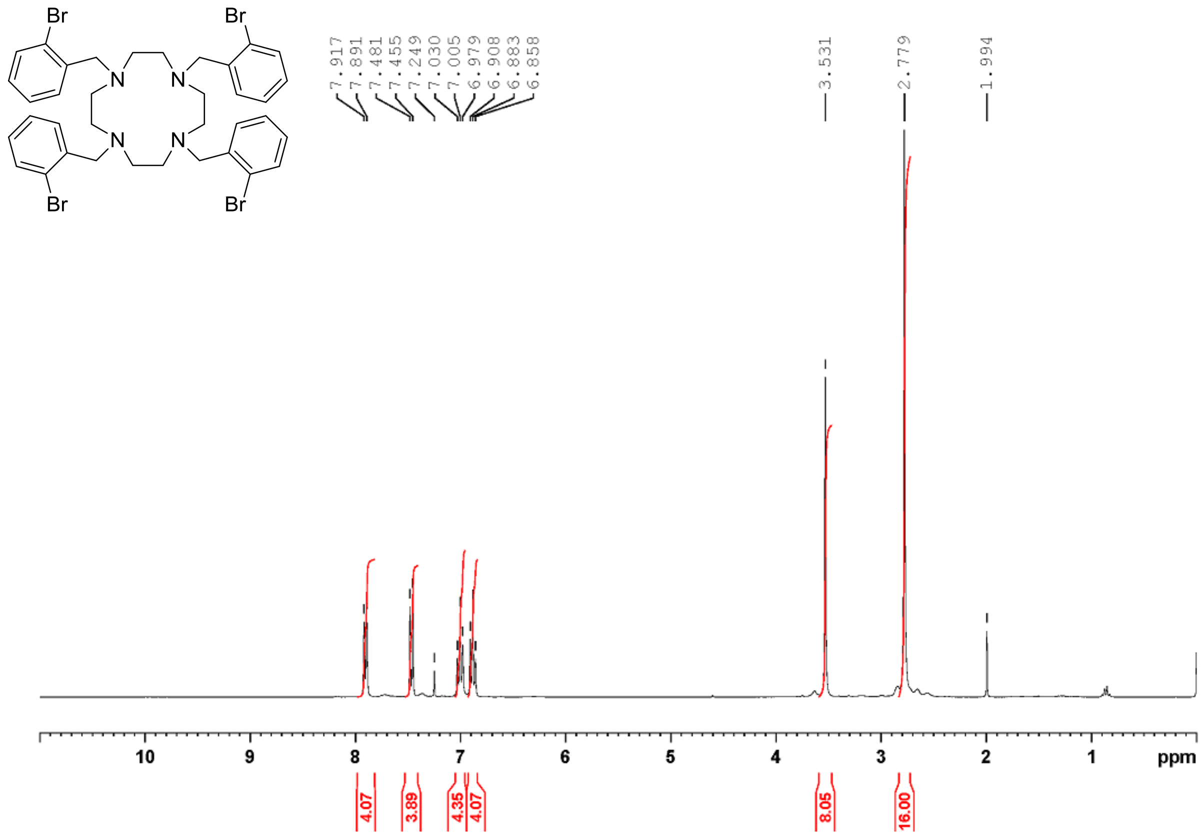

Figure S5 - ^{1}H NMR spectrum (300 MHz) of **10** in $CDCl_3$

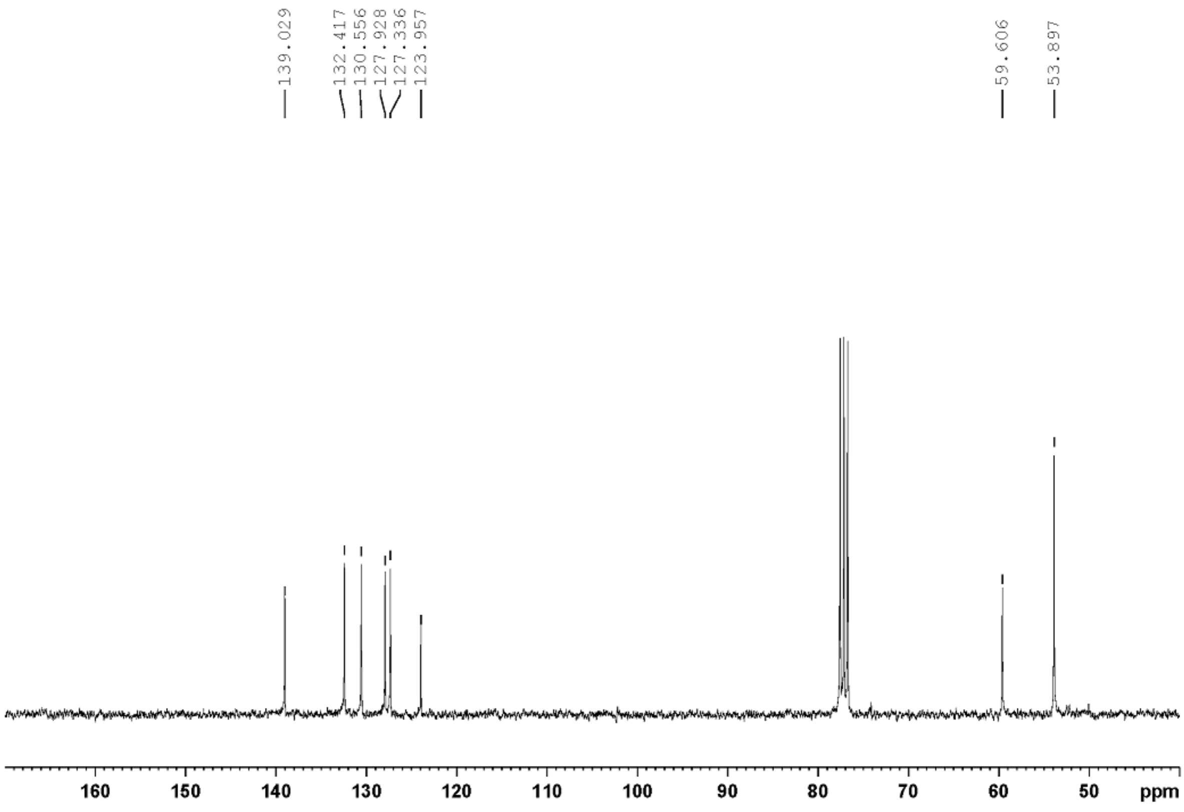

Figure S6 - ^{13}C NMR spectrum (75 MHz) of **10** in $CDCl_3$.

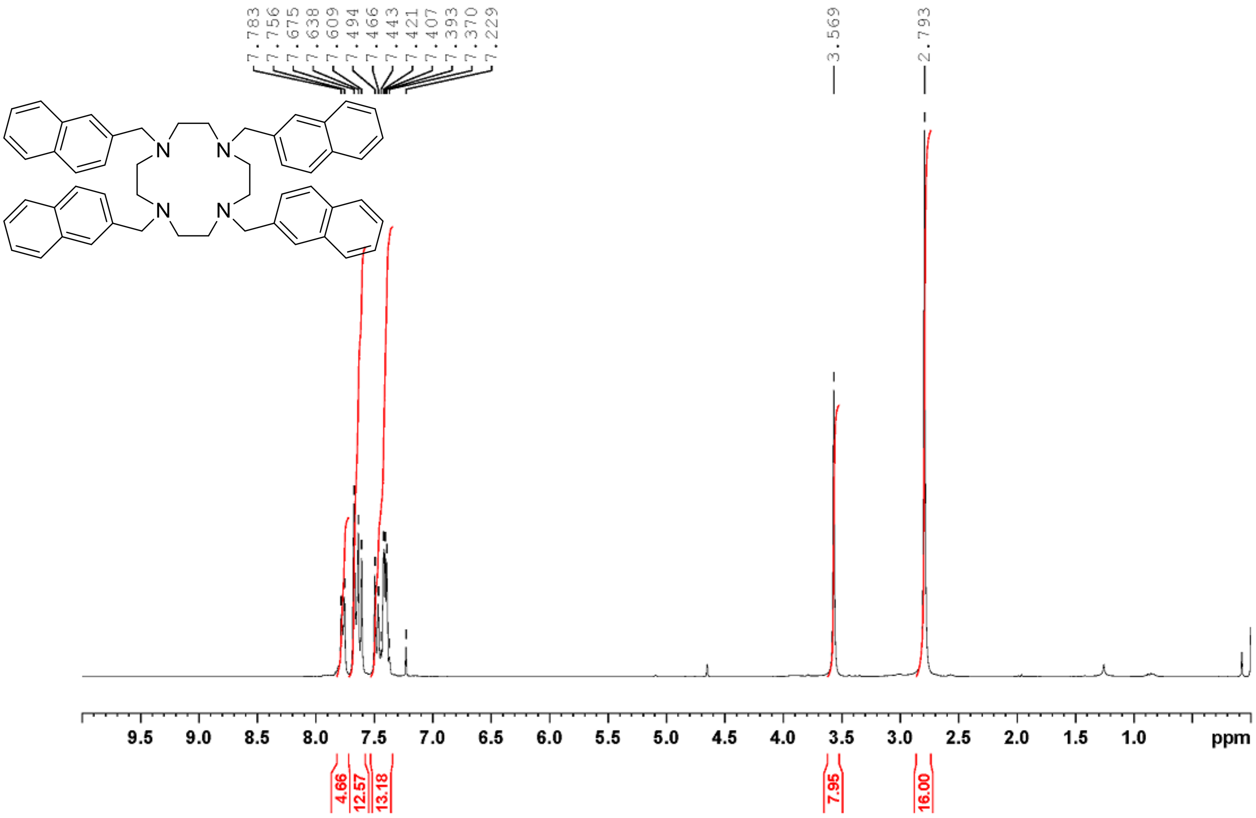

Figure S7 – ^{1}H NMR spectrum (300 MHz) of **12** in $CDCl_3$

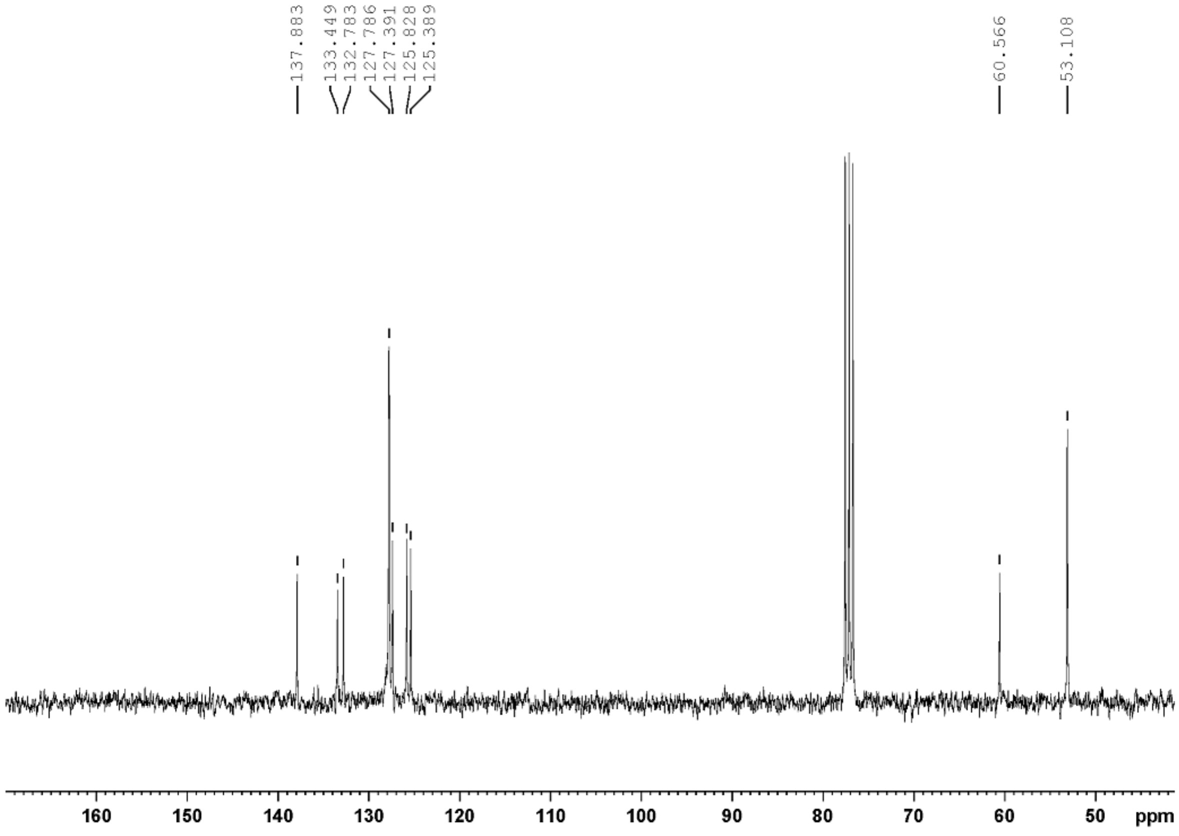

Figure S8 - ^{13}C NMR spectrum (75 MHz) of **12** in $CDCl_3$.
